# Influence of particle size and origin of field peas on apparent ileal digestibility of starch and amino acids and standardized ileal digestibility of amino acids when fed to growing pigs

**DOI:** 10.1093/tas/txae008

**Published:** 2024-02-10

**Authors:** Jimena A Ibagon, Su A Lee, C Martin Nyachoti, Hans H Stein

**Affiliations:** Department of Animal Sciences, University of Illinois, Urbana, IL 61801, USA; Department of Animal Sciences, University of Illinois, Urbana, IL 61801, USA; Department of Animal Science, University of Manitoba, Winnipeg, MB, Canada R3T 2N2; Department of Animal Sciences, University of Illinois, Urbana, IL 61801, USA

**Keywords:** field peas, ileal digestibility, particle size, pigs, starch digestibility

## Abstract

The objective was to test the hypothesis that particle size and origin of field peas influence the apparent ileal digestibility (**AID**) of starch, crude protein (**CP**), and amino acids (**AA**) and the standardized ileal digestibility (**SID**) of AA. Three sources of field peas were procured. One source was from the United States and two sources were from Canada. The U.S. source and one of the sources from Canada (i.e., Canada 1) were each divided into two batches and ground to achieve a target particle size of 250 or 450 µm, whereas the other source from Canada (i.e., Canada 2) was only ground to a target particle size of 250 µm. Each batch of field peas was included in one diet as the only source of AA. An N-free diet was used to determine the basal endogenous losses of CP and AA. Six barrows (initial weight: 50.5 kg; SD = 3.7) that had a T-cannula installed in the distal ileum were randomly allotted to a 6 × 6 Latin square design with six diets and six 7-d periods. Ileal digesta from pigs were collected for 2 d after 5 d of adaptation. Data were analyzed using a statistical model that included batch of field peas as the fixed effect and animal and period as the random effects. Contrast statements were used to analyze the effects of particle size, origin, and the interaction between particle size and origin. Results indicated that the AID of starch was increased by reducing the particle size in the U.S. source of field peas, but that was not the case for the Canada 1 source (interaction; *P* < 0.001). Particle size did not influence the AID of CP or AA, but the Canada 2 source of field peas had greater (*P* < 0.05) AID of Trp, Ala, Cys, Gly, and Tyr than the field peas from the United States. The SID of CP and AA was also not affected by the particle size of field peas. The SID of CP and Trp was greater (*P* < 0.05), and the SID of His, Ile, and Thr tended (*P* < 0.10) to be greater in the Canada 2 source compared with the U.S. source, but no differences between the two Canada sources were observed. In conclusion, a few differences in SID of AA between field peas produced in the United States and peas produced in Canada were observed, but there was no effect on SID of AA of reducing the particle size of field peas from 450 to 250 µm, whereas the AID of starch increased by reducing the particle size only in the field peas from the United States.

## Introduction

Market opportunities for field peas (*Pisum sativum* L.) have increased for livestock feed and human food, due to the high nutritional quality of pea protein (Stein et al., 2004). The nutritional value of field peas and their inclusion in corn-based diets fed to swine has been reported (Stein et al., 2006; Stein and Bohlke, 2007; Montoya and Leterme, 2011; Hugman et al., 2021). However, as is the case with most feed ingredients, differences in soil, varieties, agronomic practices, and growing conditions may affect the nutritional characteristics of the peas as well as the digestibility of nutrients (Stein et al., 2004). Differences in particle size of field peas may also change the digestibility of energy and nutrients as has been reported for other ingredients (Kim et al., 2009; Rojas and Stein, 2015, 2017; Lancheros et al., 2020). An increase in energy digestibility was also observed by reducing the particle size of field peas, which was attributed to an increase in in vitro digestibility of starch (Montoya and Leterme, 2011). There is, however, to the best of our knowledge, no information about the effects of particle size of peas on in vivo digestibility of starch and amino acids (**AA**). It is also not known if the growing location of field peas influences the digestibility of AA and starch. Therefore, this research was conducted to test the hypothesis that the apparent ileal digestibility (**AID**) of crude protein (**CP**), AA, and starch, as well as the standardized ileal digestibility (**SID**) of CP and AA in field peas, is affected by particle size and the location where the field peas were grown.

## Materials and Methods

The protocol for this experiment was reviewed and approved by the Institutional Animal Care and Use Committee at the University of Illinois.

### Experimental Diets

Three sources of field peas were procured. One source was obtained from the United States (U.S. field peas), and the other two sources (CDC Amarillo Yellow and CDC Meadow Yellow) were obtained from Canada (i.e., Canada 1, Canada 2). The field peas from the United States and the Canada 1 source were each divided into two batches and ground to ~250 or 450 µm, but due to a limited supply of peas, the Canada 2 source was only ground to a target particle size of 250 µm. Therefore, five batches of field peas were used in the experiment (Table 1). Each batch was included in one diet as the sole source of AA. A nitrogen-free (N-free) diet that was used to calculate basal endogenous losses of AA and CP was also formulated. Thus, a total of six diets were formulated (Tables 2 and 3). Vitamins and minerals were included in all diets to meet or exceed current requirement estimates for growing pigs (NRC, 2012). All diets contained 0.40% chromic oxide as an indigestible marker. The daily feed allowance was calculated as 3.0 times the maintenance requirement for metabolizable energy (i.e., 197 kcal metabolizable energy per kg body weight^0.60^; NRC, 2012). Feed allowance was adjusted according to the body weight of pigs at the beginning of each period, and water was available at all times.

**Table 1.** Analyzed nutrient composition of the five batches of field peas^1^

Item, %	Measured particle size of field peas, µm				
	265	220	253	457	411
	United States	Canada 1	Canada 2	United States	Canada 1
Gross energy, kcal/kg	3,919	3,933	3,925	3,913	3,923
Dry matter	89.54	89.99	89.72	89.21	89.79
Crude protein	19.90	19.52	20.03	19.63	19.84
Ash	2.83	2.55	2.59	2.80	2.61
Starch	38.62	40.88	39.23	40.29	42.12
Acid-hydrolyzed ether extract	0.93	1.00	1.03	0.95	1.00
Insoluble dietary fiber	15.53	17.11	15.89	15.84	16.72
Soluble dietary fiber	1.87	2.54	1.37	1.82	1.95
Total dietary fiber	17.40	19.66	17.26	17.66	18.67
Sucrose	2.65	2.90	1.72	2.51	3.05
Maltose	1.94	1.60	2.01	1.90	1.71
Stachyose	2.39	2.82	2.45	2.33	2.82
Raffinose	0.63	0.59	0.57	0.64	0.60
Indispensable AA					
Arg	1.59	1.60	1.64	1.61	1.62
His	0.49	0.51	0.51	0.50	0.50
Ile	0.93	0.98	0.93	0.93	0.94
Leu	1.44	1.51	1.50	1.45	1.50
Lys	1.54	1.58	1.59	1.54	1.59
Met	0.21	0.20	0.21	0.21	0.21
Phe	1.03	1.05	1.05	1.03	1.05
Thr	0.72	0.74	0.77	0.73	0.77
Trp	0.17	0.18	0.18	0.17	0.19
Val	0.99	1.03	1.00	1.00	1.00
Total Indispensable AA	9.12	9.37	9.38	9.18	9.35
Dispensable AA					
Ala	0.88	0.90	0.90	0.88	0.90
Asp	2.26	2.35	2.37	2.27	2.36
Cys	0.33	0.30	0.30	0.32	0.31
Glu	3.34	3.37	3.41	3.37	3.39
Gly	0.91	0.93	0.93	0.91	0.93
Pro	0.80	0.81	0.82	0.83	0.82
Ser	0.85	0.85	0.91	0.85	0.90
Tyr	0.62	0.58	0.64	0.62	0.61
Total dispensable AA	19.12	19.46	19.68	19.23	19.56
Total AA	28.24	28.83	29.05	28.41	28.91
Lys:CP^2^	7.62	7.98	7.80	7.73	7.87

^1^All values except dry matter are expressed on an 88% dry matter basis. Peas were ground to a target particle size of 250 or 450.

^2^Lys:CP ratio was calculated by expressing the concentration of Lys in each source of field peas as a percentage of the concentration of CP (Almeida et al., 2013).

**Table 2.** Ingredient composition of experimental diets

Item, %	Field peas					N-free
	Particle size^1^: 250 µm			450 µm		
	United States	Canada 1	Canada 2	United States	Canada 1	
Field peas	77.90	77.90	77.90	77.90	77.90	-
Corn starch	-	-	-	-	-	72.65
Soybean oil	4.00	4.00	4.00	4.00	4.00	4.00
Solka floc^2^	-	-	-	-	-	4.00
Dicalcium phosphate	0.70	0.70	0.70	0.70	0.70	2.10
Ground limestone	1.10	1.10	1.10	1.10	1.10	0.45
Sucrose	15.0	15.0	15.0	15.0	15.0	15.0
Sodium chloride	0.40	0.40	0.40	0.40	0.40	0.40
Vitamin-mineral premix^3^	0.50	0.50	0.50	0.50	0.50	0.50
Chromic oxide	0.40	0.40	0.40	0.40	0.40	0.40
Potassium carbonate	-	-	-	-	-	0.40
Magnesium oxide	-	-	-	-	-	0.10

^1^The actual measured particle size of field peas ground to 250 µm was 265, 220, and 253 µm in the United States, Canada 1, and Canada 2 sources, respectively, and the measured particle size of the United States and Canada 1 peas ground to 450 µm was 457, and 411 µm, respectively.

^2^Fiber Sales and Development Corp., Urbana, OH, USA.

^3^The vitamin-micromineral premix provided the following quantities of vitamins and micro minerals per kg of complete diet: vitamin A as retinyl acetate, 10,622 IU; vitamin D_3_ as cholecalciferol, 1,660 IU; vitamin E as DL alpha-tocopheryl acetate, 66 IU; vitamin K as menadione nicotinamide bisulfate, 1.40 mg; thiamin as thiamine mononitrate, 1.08 mg; riboflavin, 6.49 mg; pyridoxine as pyridoxine hydrochloride, 0.98 mg; vitamin B_12_, 0.03 mg; _D-_pantothenic acid as _D-_calcium pantothenate, 23.2 mg; niacin, 43.4 mg; folic acid, 1.56 mg; biotin, 0.44 mg; Cu, 20 mg as copper chloride; Fe, 123 mg as iron sulfate; I, 1.24 mg as ethylenediamine dihydriodide; Mn, 59.4 mg as manganese hydroxychloride; Se, 0.27 mg as sodium selenite and selenium yeast; and Zn, 124.7 mg as zinc hydroxychloride.

**Table 3.** Analyzed nutrient composition of experimental diets, as fed basis

Item, %	Field peas					N-free
	Particle size^1^: 250 µm			450 µm		
	United States	Canada 1	Canada 2	United States	Canada 1	
Dry matter	89.98	89.13	89.03	89.96	89.20	91.10
Crude protein	14.62	14.87	15.14	15.22	14.99	0.14
Starch	31.4	31.3	31.1	30.1	31.1	62.9
Indispensable amino acids						
Arg	1.22	1.16	1.19	1.13	1.18	0.01
His	0.38	0.37	0.37	0.36	0.37	-
Ile	0.72	0.68	0.70	0.69	0.69	0.01
Leu	1.16	1.10	1.13	1.12	1.1	0.02
Lys	1.18	1.09	1.15	1.11	1.11	0.01
Met	0.17	0.16	0.16	0.15	0.16	-
Phe	0.80	0.75	0.78	0.78	0.76	0.01
Thr	0.57	0.55	0.56	0.54	0.55	0.01
Trp	0.12	0.12	0.12	0.12	0.14	<0.02
Val	0.76	0.73	0.76	0.72	0.74	0.01
Total indispensable Amino aicds	7.08	6.71	6.92	6.72	6.80	0.08
Dispensable amino acids						
Ala	0.7	0.68	0.68	0.67	0.68	0.01
Asp	1.72	1.64	1.73	1.66	1.66	0.02
Cys	0.24	0.21	0.22	0.21	0.23	-
Glu	2.61	2.47	2.51	2.55	2.54	0.04
Gly	0.7	0.68	0.69	0.67	0.68	0.01
Pro	0.66	0.63	0.61	0.63	0.63	0.01
Ser	0.67	0.64	0.66	0.65	0.64	0.01
Tyr	0.48	0.46	0.48	0.45	0.47	0.01
Total dispensable Amino acids	7.78	7.41	7.58	7.49	7.53	0.10
Total amino acids	15.11	14.38	14.73	14.47	14.57	0.37

^1^The actual measured particle size of field peas ground to 250 µm was 265, 220, and 253 µm in the United States, Canada 1, and Canada 2 sources, respectively, and the measured particle size of the United States and Canada 1 peas ground to 450 µm was 457, and 411 µm, respectively.

### Animals and Housing

Six growing pigs with an average initial body weight of 50.5 ± 3.7 kg had a T-cannula installed in the distal ileum (Stein et al., 1998). Pigs were the offspring of Line 359 males mated to Camborough females (Pig Improvement Company, Hendersonville, TN, USA) and were allotted to a 6 × 6 Latin square design with six diets and six periods (Kim and Stein, 2009). Pigs were individually housed in pens (1.2 × 1.5 m) located in an environmentally controlled room with the ambient temperature maintained between 20 and 24 °C. Pens had smooth sidings and fully slatted tribar floors, and a feeder and a water nipple were installed in each pen.

### Sample Collection

Each period of the Latin square lasted 7 days, with the initial 5 days being the adaptation period to the diet, whereas ileal digesta were collected on days 6 and 7 for 9 hours each day (Stein et al., 1998). By attaching a plastic bag to the opened cannula barrel using a cable tie, digesta that flowed into the bag were collected. Bags were replaced every time they were filled with digesta or at least once every 30 min. Digesta samples were immediately stored at −20 °C to prevent bacterial degradation of AA.

### Chemical Analysis

One sample of each diet and of each batch of field peas was collected at the time of diet mixing and stored for later analysis. At the conclusion of the animal part of the experiment, ileal digesta samples were thawed at room temperature and mixed within animal and diet. A sub-sample was collected, lyophilized, ground, and analyzed. All samples were analyzed in duplicate. The concentration of chromium was determined in diets and ileal digesta using the Inductive Coupled Plasma Atomic Emission Spectrometric method (method 990.08; AOAC Int., 2019). Samples were prepared for analysis using nitric acid-per-chloric acid [method 968.08D(b); AOAC Int., 2019]. Diets, ingredients, and ileal digesta samples were analyzed for dry matter via oven drying at 135 °C for 2 hours (method 930.15; AOAC Int., 2019) and ingredients were also analyzed for dry ash (method 942.05; AOAC Int., 2019). Nitrogen in ingredients, diets, and in the ileal digesta samples was determined by the combustion procedure using a LECO FP628 Nitrogen Analyzer (LECO Corp., St. Joseph, MI, USA; method 990.03; AOAC Int., 2019) and CP was calculated as analyzed nitrogen × 6.25. Ingredients, diets, and ileal digesta samples were also analyzed for AA [method 982.30 E(a, b, c); AOAC Int., 2019] on a Hitachi Amino Acid Analyzer, Model No. L8800 (Hitachi High Technologies America, Inc.; Pleasanton, CA, USA) using ninhydrin for post-column derivatization and nor-leucine as the internal standard.

Gross energy in ingredient samples was measured using an isoperibol bomb calorimeter (Model 6400, Parr Instruments, Moline, IL, USA). Benzoic acid was used as the standard for calibration. Ingredients were also analyzed for acid-hydrolyzed ether extract using the acid hydrolysis filter bag technique (Ankom HCl Hydrolysis System; Ankom Technology, Macedon, NY, USA) followed by crude fat extraction using petroleum ether (method 2003.06, AOAC Int., 2019) in an AnkomXT^15^ Extractor (Ankom Technology). Insoluble dietary fiber and soluble dietary fiber were analyzed in ingredients according to method 991.43 (AOAC Int., 2019) using the Ankom^TDF^ Fiber Analyzer (Ankom Technology). Total dietary fiber was calculated as the sum of soluble dietary fiber and insoluble dietary fiber. Ingredient samples were also analyzed for sugars, including maltose, sucrose, stachyose, and raffinose, using high-performance liquid chromatography (Dionex App Notes 21 and 92). Ingredients, diets, and ileal digesta samples were analyzed for total starch by the glucoamylase procedure (method 979.10; AOAC Int., 2019). Particle size of field peas was determined using 100 g of the ingredient that was placed on top of test sieves and placed in a vibratory sieve shaker for 15 min. The weight of the field pea material in each of the test sieves was recorded for the calculation of mean particle size (ANSI/ASAE, 2008).

### Calculations and Statistical Analysis

The AID of CP, AA, and starch in diets was calculated from analyzed concentrations of CP, AA, starch, and Cr in diets and ileal digesta (Stein et al., 2007). The basal endogenous losses of CP and AA were calculated from pigs fed the N-free diet and the SID of CP and AA was calculated by correcting AID values for basal endogenous losses of CP and AA (Stein et al., 2007). Because field peas were the sole source of CP and AA in each diet, values for AID and SID in diets were considered the AID or SID of field peas.

Data were analyzed using the PROC MIXED procedure (SAS Inst. Inc., Cary, NC, USA). Normality of residuals was confirmed using the MIXED procedure and homogeneity of the variance of the residuals was tested using Brown-Forsythe test in the GLM procedure of SAS. The statistical model included field pea batch as fixed effect and period and animal as random effects. Preplanned contrast statements were used to compare results for field peas ground to 250 µm with results for field peas ground to 450 µm, the origin of the source, and the interaction between the source and the particle size. Results were considered significant at *P* ≤ 0.05. The pig was the experimental unit for all analyses.

## RESULTS

The gross energy among all sources of field peas ranged from 3,913 to 3,933 kcal/kg, and the CP ranged from 17.86% to 19.81% (Table 1). Values for acid-hydrolyzed ether extract varied between 0.90% and 1.03%. The concentration of total dietary fiber in peas from the United States and one of the sources from Canada was around 17.65%, but for the other source from Canada, total dietary fiber was 20.10%. The two sources of field peas from Canada had the numerically greatest concentrations of all AA and also the greatest Lys:CP ratio. All sources of peas contained around 40% starch.

The AID of CP was greater (*P* < 0.05) in the two Canadian sources of peas than in the U.S. peas (Table 4). When ground to 250 µm, no differences among sources were observed for the AID of starch, but the U.S. field peas ground to 450 µm had reduced AID of starch compared with the Canada 1 source ground to 450 µm (interaction *P* < 0.001). There were no differences in the AID of indispensable AA among the five batches of peas with the exception that Trp had a lower (*P* < 0.05) AID in the U.S. field peas than in the Canada 1 source of peas. Among dispensable AA, the AID of Ala, Cys, Gly, and Tyr was less (*P* < 0.05) in the U.S. peas compared with both Canadian sources. The AID of Glu was lower (*P* < 0.05) when field peas were ground to 250 µm compared with field peas ground to 450 µm.

**Table 4.** Apparent ileal digestibility of crude protein, starch, and amino acids (AA) %, in three sources of field peas ground to two particle sizes^1^

Item, %	Field peas					SEM	Contrast *P*-value		
	Particle size^2^:250 µm			450 µm					
	Source: United States	Canada 1	Canada 2	United States	Canada 1		Particle size	Source	Interaction
Crude protein	63.32	69.45	72.16	67.75	72.83	2.38	0.326	0.017	0.809
Starch	87.33	85.08	85.91	78.90	85.20	1.28	<0.001	0.061	<0.001
Indispensable AA									
Arg	84.45	84.81	87.39	85.45	87.35	1.40	0.409	0.318	0.497
His	75.50	78.38	80.08	78.32	80.93	1.78	0.241	0.079	0.929
Ile	69.41	71.98	74.48	71.68	75.69	2.30	0.342	0.106	0.716
Leu	70.30	73.41	76.33	74.29	77.05	2.30	0.208	0.148	0.929
Lys	78.72	80.93	83.12	80.33	83.25	1.87	0.566	0.129	0.829
Met	69.90	73.45	75.34	70.05	75.12	2.78	0.895	0.104	0.767
Phe	71.44	73.83	76.78	75.44	77.89	2.10	0.109	0.178	0.988
Thr	62.79	67.42	69.68	64.79	70.13	2.83	0.729	0.068	0.893
Trp	62.99	67.91	69.80	66.09	74.72	2.84	0.172	0.021	0.502
Val	66.27	69.52	72.89	69.44	73.50	2.57	0.355	0.113	0.859
Average	73.29	75.77	78.37	75.72	78.98	2.05	0.344	0.115	0.827
Dispensable AA									
Ala	64.55	69.18	71.58	65.66	71.91	2.63	0.874	0.034	0.738
Asp	72.20	74.38	77.42	74.23	77.47	1.98	0.425	0.104	0.744
Cys	54.07	56.21	59.80	48.89	61.81	3.42	0.654	0.030	0.110
Glu	77.42	79.49	80.80	81.60	82.85	1.86	0.029	0.242	0.766
Gly	53.93	59.45	62.13	54.30	63.61	3.83	0.884	0.041	0.578
Ser	69.59	72.45	74.80	72.01	75.49	1.97	0.369	0.084	0.863
Tyr	73.27	76.65	78.79	74.53	79.29	1.98	0.675	0.029	0.693
Average	70.78	73.63	75.84	73.22	76.95	2.08	0.291	0.065	0.797
Average, all AA	72.03	74.70	77.10	74.46	77.96	2.05	0.312	0.083	0.808

^1^Each least squares mean is the mean of five observations per treatment.

^2^The actual measured particle size of field peas ground to 250 µm was 265, 220, and 253 µm in the United States, Canada 1, and Canada 2 sources, respectively, and the measured particle size of the United States and Canada 1 peas ground to 450 µm was 457, and 411 µm, respectively.

The SID of CP was greater (*P* < 0.05) in both Canadian sources of field peas than in U.S. peas (Table 5). No interaction between particle size and source of field peas was observed for the SID of AA. The SID of indispensable AA was not different among sources of peas, but the SID of Ala, Cys, Gly, and Tyr was greater in field peas from Canada than in peas from the United States. Reduction of the particle size from 450 to 250 did not impact SID of CP or AA.

**Table 5.** Standardized ileal digestibility of crude protein and amino acids (AA), % in three sources of field peas ground at two particle sizes^1,2^

Item, %	Field peas					SEM	Contrast *P*-value		
	Particle size^3^: 250 µm			450 µm					
	United States	Canada 1	Canada 2	United States.	Canada 1		Particle size	Source	Interaction
Crude protein	73.88	79.73	82.25	77.88	83.03	2.4	0.360	0.019	0.872
Indispensable AA									
Arg	90.05	90.65	93.08	91.50	93.09	1.4	0.316	0.333	0.659
His	80.49	83.45	85.15	83.58	86.00	1.8	0.208	0.084	0.858
Ile	73.95	76.75	79.11	76.43	80.39	2.3	0.321	0.098	0.766
Leu	74.58	77.89	80.68	78.73	81.54	2.3	0.192	0.134	0.900
Lys	82.02	84.46	86.46	83.83	86.72	1.9	0.524	0.115	0.893
Met	74.40	78.18	80.06	75.15	79.85	2.8	0.984	0.109	0.857
Phe	75.39	78.00	80.79	79.49	82.01	2.1	0.104	0.154	0.978
Thr	71.10	75.96	78.06	73.56	78.67	2.8	0.652	0.068	0.962
Trp	70.33	75.18	77.06	73.42	80.96	2.8	0.239	0.033	0.627
Val	71.71	75.14	78.27	75.18	79.04	2.6	0.316	0.115	0.924
Average	78.17	80.87	83.31	80.86	84.01	2.1	0.310	0.108	0.899
Dispensable AA									
Ala	73.43	78.23	80.63	74.94	80.97	2.6	0.812	0.034	0.799
Asp	76.64	78.99	81.78	78.83	82.03	2.0	0.386	0.096	0.794
Cys	61.64	64.78	67.97	57.54	69.64	3.4	0.687	0.028	0.180
Glu	81.08	83.32	84.56	85.35	86.58	1.9	0.030	0.222	0.715
Gly	78.64	84.64	86.93	80.11	88.82	3.8	0.732	0.042	0.690
Ser	76.59	79.71	81.84	79.22	82.75	2.0	0.326	0.071	0.910
Tyr	77.91	81.45	83.38	79.48	83.99	2.0	0.609	0.031	0.780
Average	78.17	80.87	83.31	80.86	84.01	2.1	0.310	0.108	0.899
Average, all AA	77.72	80.85	82.86	80.42	84.05	2.1	0.265	0.058	0.884

^1^Each least squares mean is the mean of five observations per treatment.

^2^Values for SID were calculated by correcting values for AID for basal ileal endogenous losses. Basal ileal endogenous losses were determined (g/kg of dry matter intake) as CP, 14.54; Arg, 0.66; His, 0.18; Ile, 0.32; Leu, 0.48; Lys, 0.38; Met, 0.07; Phe, 0.31; Thr, 0.46; Trp, 0.09; Val, 0.40; Ala, 0.60; Asp, 0.74; Cys, 0.18; Glu, 0.93; Gly, 1.68; Ser, 0.46; and Tyr, 0.22.

^3^The actual measured particle size of field peas ground to 250 µm was 265, 220, and 253 µm in the United States, Canada 1, and Canada 2 sources, respectively, and the measured particle size of the United States and Canada 1 peas ground to 450 µm was 457, and 411 µm, respectively.

## DISCUSSION

Field peas may be cultivated in climates that are too cold for cultivation of soybeans including areas of Europe, Canada, and the Pacific Northwest of the United States (Jezierny et al., 2010). The global production of dry peas is ~14 million metric tonnes per year and the majority of peas are grown for human consumption. The major producers, including Canada, Russia, China, and the United States, have expanded production of field peas by ~10% in recent years (FAO, 2022). Because of their high-quality protein and carbohydrate content, field peas are an excellent ingredient in pig diets (Stein et al., 2004), and inclusion of field peas in diets for pigs, when markets are favorable, may lower production costs.

The field peas used in this experiment originated from different varieties. Some of the variability in nutrient composition among pulses may be related to differences in growing regions, climate, and varieties (Lu et al., 2020; Abdulla et al., 2021). However, the gross energy of field peas used in this experiment was in agreement with values reported for different varieties (Stein et al., 2010; NRC, 2012; Landero et al., 2014; Adekoya and Adeola, 2022), but greater than the values reported by Hugman et al. (2021). Analyzed CP and AA were lower than the CP and AA reported by NRC (2012), but were within the range of values analyzed in other experiments (Stein et al., 2016; Hugman et al., 2021; Adekoya and Adeola, 2022). The variation in CP between the field peas used in this experiment and peas used in some previous experiments may be a result of differences in varieties or environmental factors because the amount of rainfall, level of fertilizers, and harvesting time may influence the chemical composition of field peas (Wang and Daun, 2004). The starch content of field peas used in this experiment was within the range of 39% to 42%, which is in agreement with values reported by Stein et al. (2016) and Ravindran et al. (2010), but greater than other reported values (Landero et al., 2014; Hugman et al., 2021; Woyengo and Zijlstra, 2021). The slightly lower value for SDF in the Canada 2 source of field peas than in the other sources may be a result of genetic differences among the three varieties used in the experiment. However, it is also possible that the difference is due to analytical inaccuracies because values for SDF are usually more variable than values for IDF.

The SID of CP and AA in the U.S. field peas ground to 250 or 450 µm was lower than some previous values (Stein et al., 2004; NRC, 2012), but both Canadian sources of field peas ground to 250 µm had SID of AA close to reported values (Stein et al., 2004; Friesen et al., 2006; NRC, 2012; Hugman et al., 2021). Digestibility of CP and AA in field peas may vary due to differences among varieties and concentrations of antinutritional factors (Leterme et al., 1990; Mariscal-Landín et al., 2002). The digestibility of AA is also influenced by the level of fiber in the diets (Mosenthin et al., 1994), but because the concentration of fiber was largely constant among diets, it is not likely that fiber concentrations contributed to the differences in digestibility of AA among sources.

The AID of starch for the field peas used in this experiment was lower than the values reported by Woyengo and Zijlstra (2021) and Hugman et al. (2021), but close to the values reported by Stein and Bohlke (2007). Processing of cereal grains, legumes, and other plant ingredients is used to maximize utilization of nutrients (Goodband et al., 2002; Rojas and Stein, 2017), and particle size reduction can modify the physical structure of feed ingredients and positively impact their nutritional characteristics (Lancheros et al., 2020). By providing a larger surface area for contact between the digestive enzymes and the substrate, grinding increases the digestibility of nutrients (Kim et al., 2002). However, grinding to a very small particle size may generate problems with flowability and management of the diets as well as ulcers and parakeratosis in pigs (Wondra et al., 1995; Rojas et al., 2016), and the smallest particle size, therefore, is not always preferable. Digestibility of starch in cereal grains and legumes is correlated with the average particle size (Montoya and Leterme, 2011), because decreasing the particle size of peas may change the anatomy of the starch granules, which may increase access of α-amylase to the starch granules, increasing the digestion of starch (Kim et al., 2002, 2009; Rojas and Stein., 2015). The observation that particle size influenced the AID of starch in only one of the sources of peas used in the current experiment indicates that the reduction from 450 to 250 µm was not sufficient to improve starch digestion in all sources of field peas. When the particle size of corn was reduced from more than 800 to 339 µm, a linear increase in AID of starch was observed (Rojas and Stein, 2015) indicating that greater variability in particle size among the sources of field peas used in this experiment may have been needed to obtain significant differences in AID of starch in both sources. It is also possible that the reason the Canada 1 source of field peas did not have increased AID of starch as particle size was reduced is that the starch in this source of field peas is different from the starch in the field peas from the United States, but because no characterization of the starch granules was accomplished in this experiment, we are not able to confirm this hypothesis.

The SID of AA and CP in cereal grains and legumes may increase as particle size decreases (Lahaye et al., 2007; Kim et al., 2009) due to greater access of enzymes to the proteins in the small intestine. However, Rojas and Stein (2015), demonstrated that a reduction in particle size of corn did not influence the digestibility of AA, and results of the present experiment indicating that the SID of AA was not increased by particle size reduction is in agreement with Rojas and Stein (2015). It is, however, possible that a greater reduction in particle size than used in this experiment would have had an impact on AA digestibility, but further research is needed to confirm this hypothesis.

## CONCLUSION

Under the conditions of this experiment, reduction in particle size of field peas from 450 to 250 µm did not influence the SID of AA and CP, but the SID of CP and some dispensable AA was greater in field peas from Canada than in field peas grown in the United States. The AID of starch increased with reduction of particle size in the U.S. source of peas, but the AID of starch in the Canadian field peas was not impacted by particle size.
